# Supplementary material for: Identification of Key Transcription Factors Related to Bacterial Spot Resistance in Pepper through Regulatory Network Analyses
Source: Genes (Basel). 2021 Aug 29;12(9):1351. doi: 10.3390/genes12091351 (PMC8472308; doi:10.3390/genes12091351)
Supplement: Supplementary file 1 [file genes-12-01351-s001.zip › genes-1333683-supplementary figures.pdf]

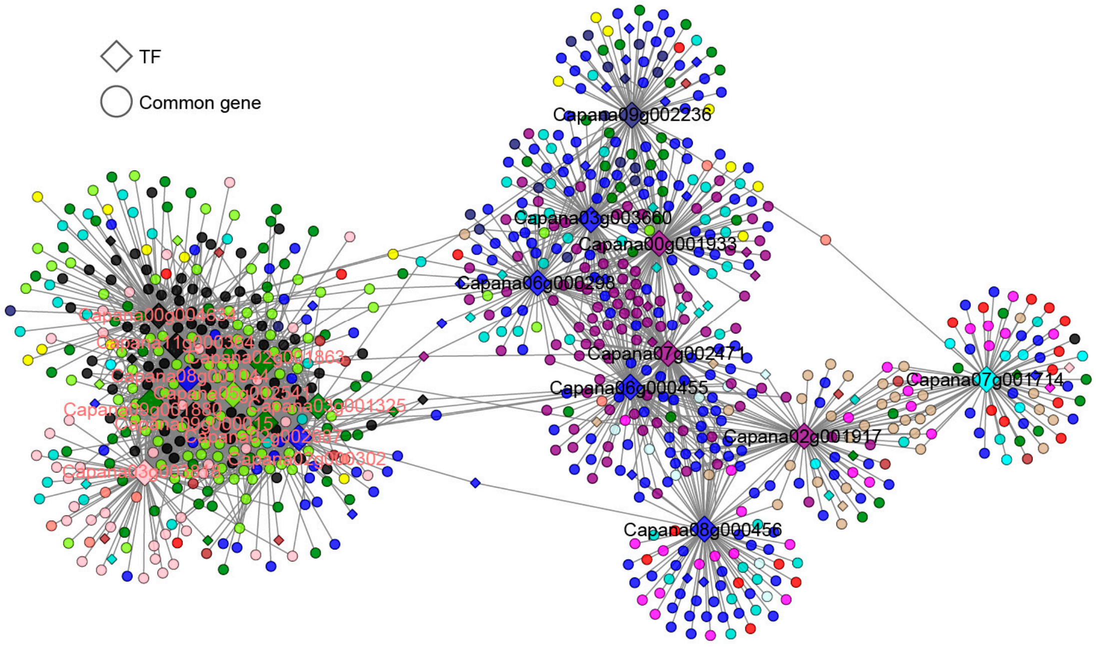

Figure S1. The transcriptional regulatory network of 20 TFs. Each node represents a gene, and genes exhibiting a regulatory relationship are connected with an edge. The diamond frames represent TFs; the circles represent other genes. Genes belonging to different co-expression modules (as determined by WGCNA) are indicated in different colors that correspond to those of co-expression modules.

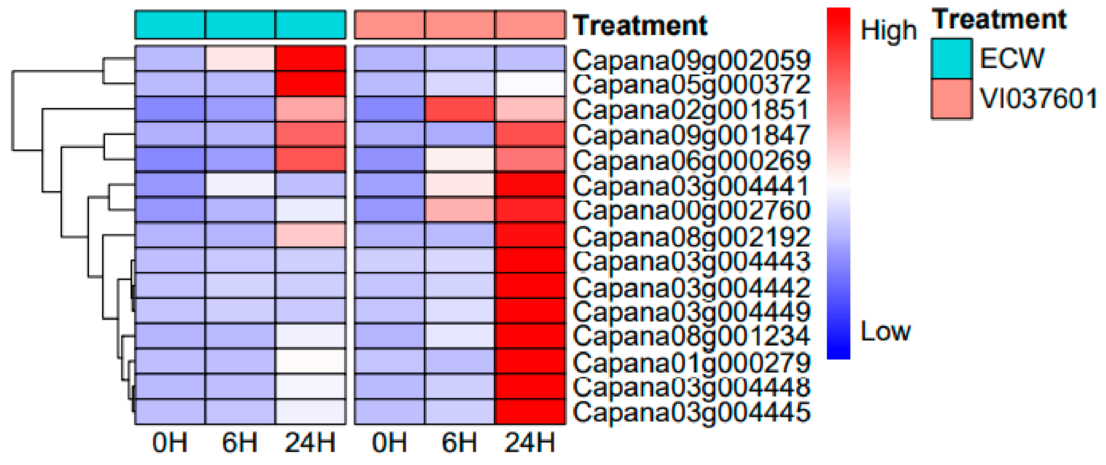

Figure S2. Heatmap of PR genes differentially expressed between ECW and VI037601 post *Xcv*

infection. The color gradient represents the normalized FPKM value (Z-score) of genes (high expression (red) and low expression (blue)).

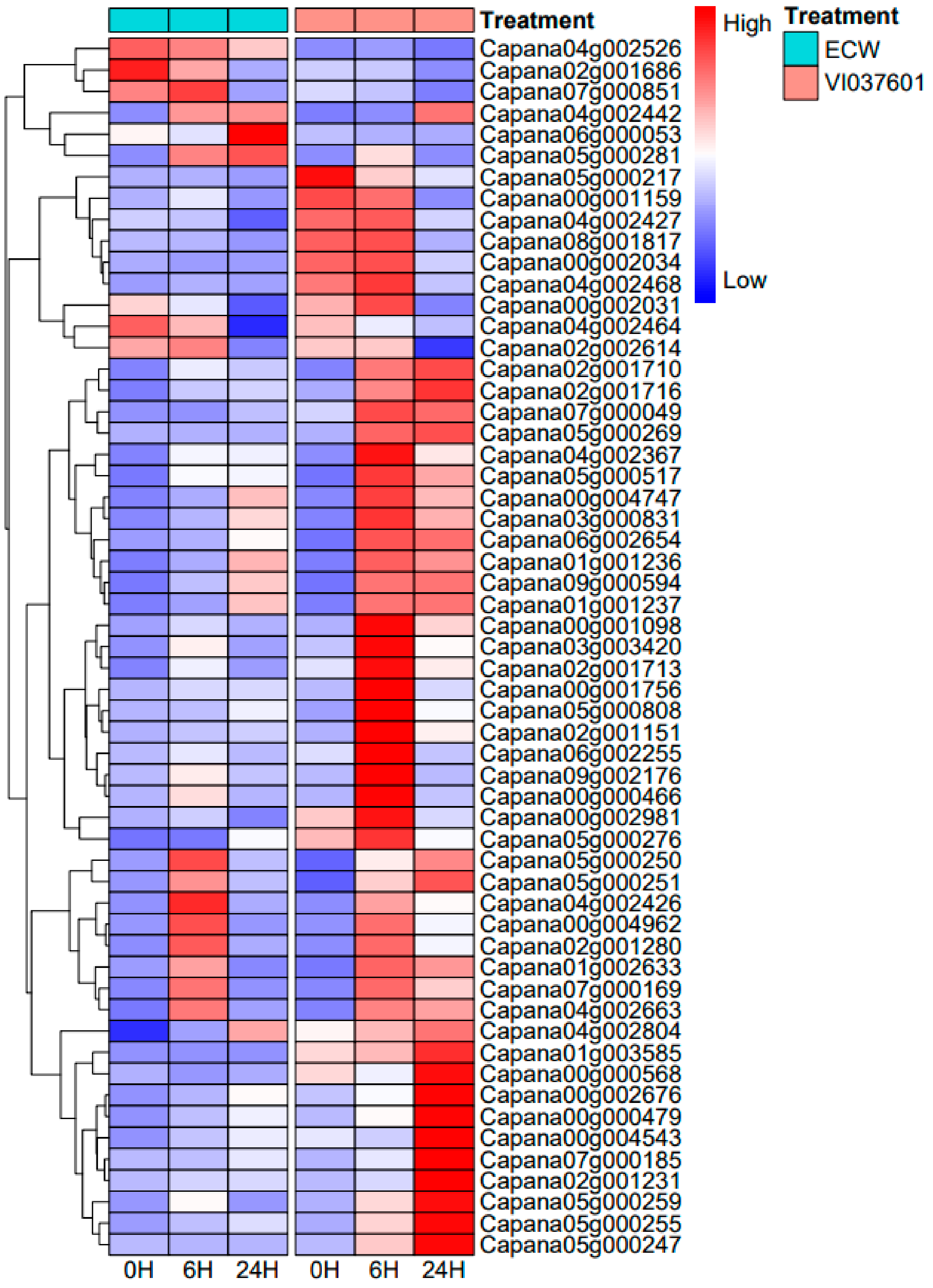

Figure S3. Heatmap of PRRs differentially expressed between ECW and VI037601 post *Xcv* in-

fection. The color gradient represents the normalized FPKM value (Z-score) of genes (high expression (red) and low expression (blue)).

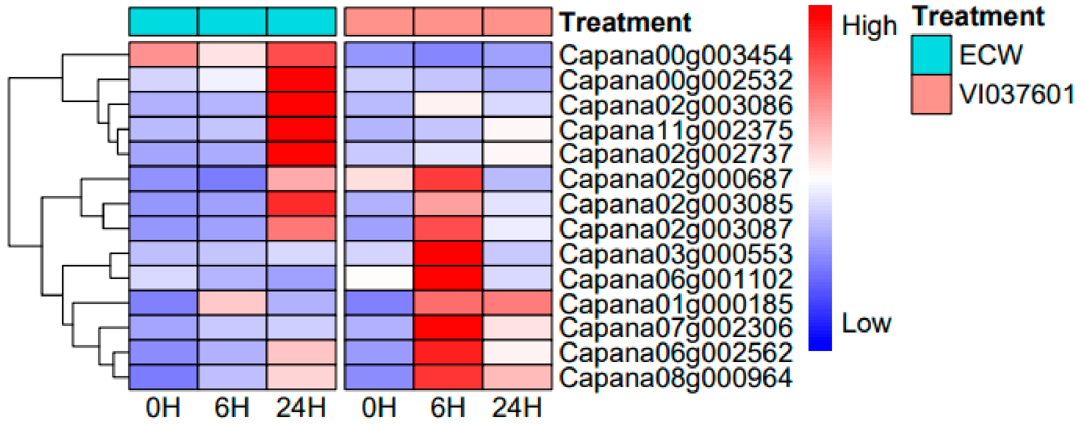

Figure S4. Heatmap of MAPKs differentially expressed between ECW and VI037601 post *Xcv* infection. The color gradient represents the normalized FPKM value (Z-score) of genes (high expression (red) and low expression (blue)).

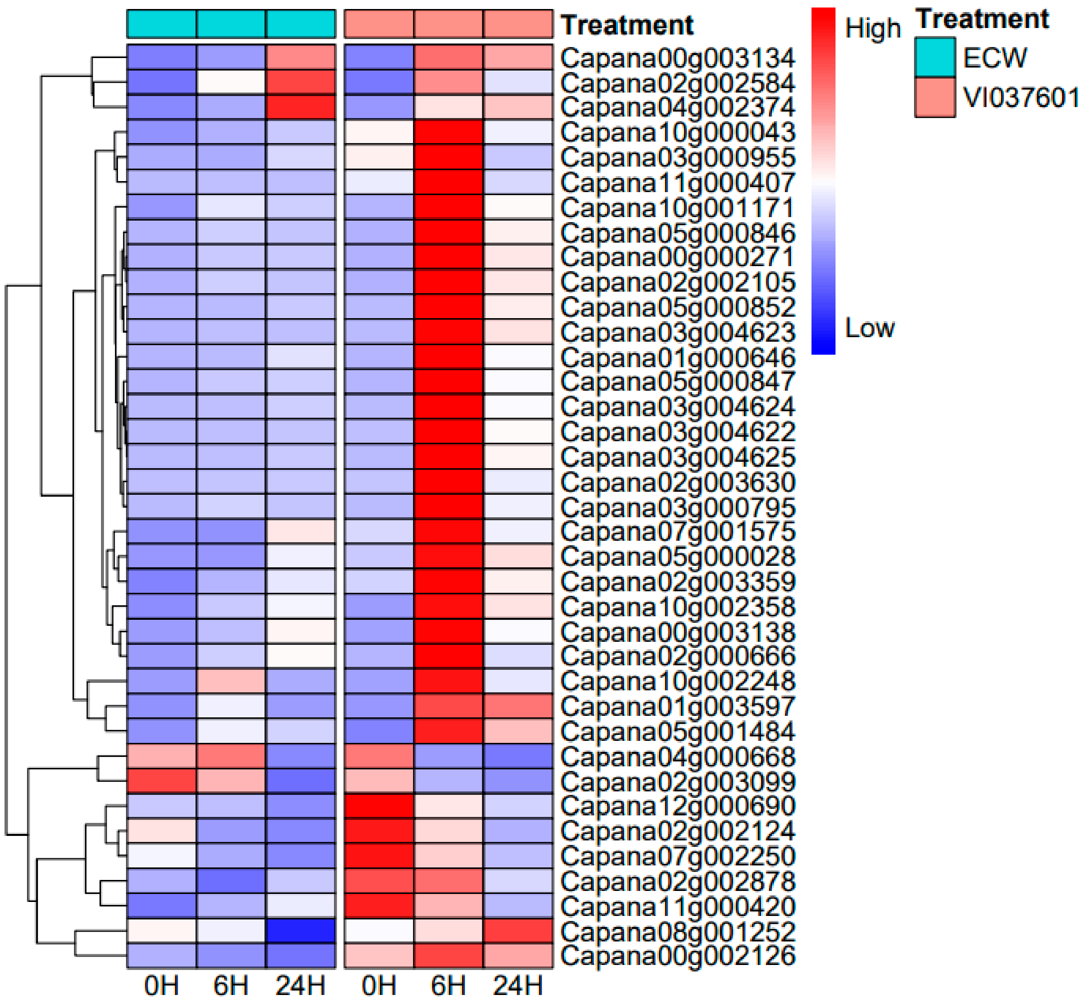

Figure S5. Heatmap of calcium signaling genes differentially expressed between ECW and VI037601 post *Xcv* infection. The color gradient represents the normalized FPKM value (Z-score) of genes (high expression (red) and low expression (blue)).

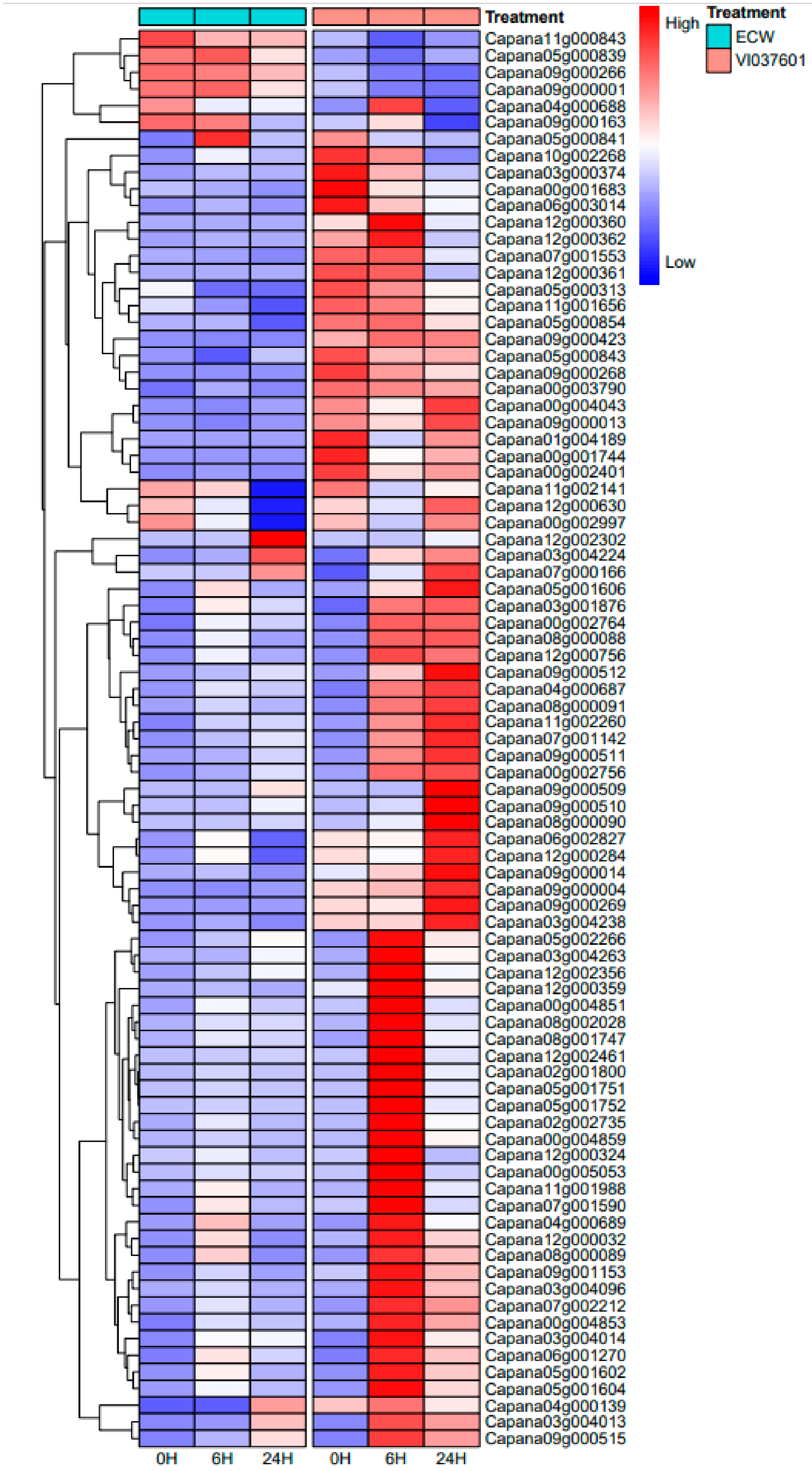

Figure S6. Heatmap of R genes differentially expressed between ECW and VI037601 post *Xcv* infection. The color gradient represents the normalized FPKM value (Z-score) of genes (high expression (red) and low expression (blue)).

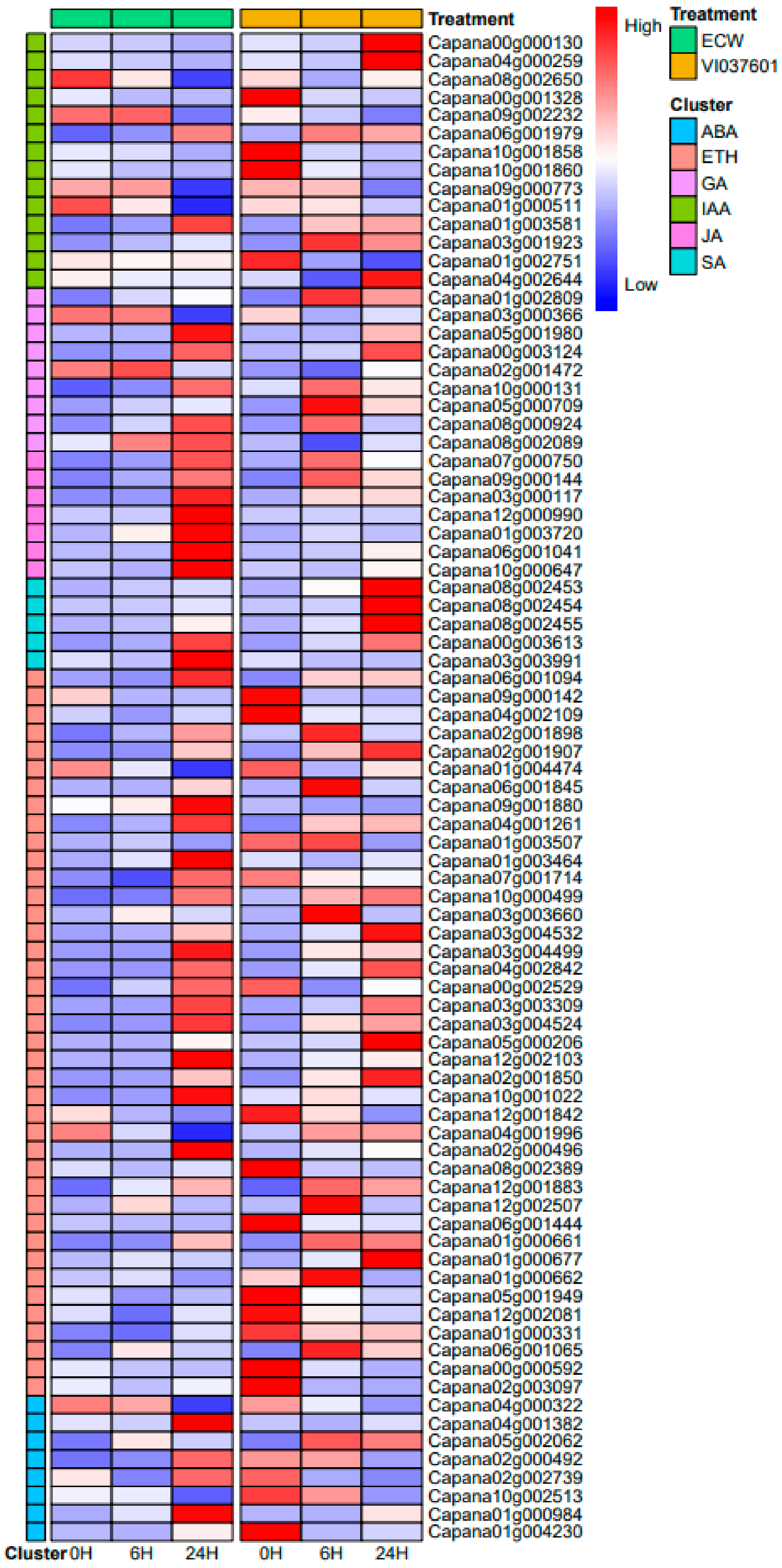

Figure S7. Heatmap of hormone-related genes differentially expressed between ECW and VI037601 post *Xcv* infection. The color gradient represents the normalized FPKM value (Z-score) of genes (high expression (red) and low expression (blue)).
