# Supplementary material for: Identification of Key Transcription Factors Related to Bacterial Spot Resistance in Pepper through Regulatory Network Analyses
Source: Genes (Basel). 2021 Aug 29;12(9):1351. doi: 10.3390/genes12091351 (PMC8472308; doi:10.3390/genes12091351)
Supplement: Supplementary file 1 [file genes-12-01351-s001.zip › genes-1333683-supplementary methods.pdf]

Methods S1. R codes for WGCNA and GENIE3.

WGCNA

```
rm(list = ls())
options(stringsAsFactors = F)

library(reshape2)
library(stringr)
library(WGCNA)
library(export)
library(gplots)
enableWGCNAThreads(nThreads = 10)

fpkm <- read.table('pepper_fpkm.txt',row.names = 1,header = T,sep = '\t')
merge <- fpkm[apply(fpkm,1,mad)> 0.5,]
merged <- t(merge)
dim(merged)
datExpr <- merged

gsg<-goodSamplesGenes(datExpr,verbose = 3)
gsg$allOK
sampletree <-hclust(dist(datExpr), method = "average")

sizeGrWindow(13,10)
par(mar=c(0,5,2,0))
plot(sampletree, main = "Sample clustering to detect outliers",sub = '',
     xlab="",cex.lab = 1.5, cex.axis=1.5, cex.main = 2.5,cex=1.5 )
dim(datExpr)

pheno <-read.csv('WGCNA/pheno-all-samples.csv',row.names = 1)
save(datExpr,pheno,file='data_input.Rdata')

powers = c(c(1:10), seq(from = 12, to=20, by=2))
sft = pickSoftThreshold(datExpr, RsquaredCut = 0.85,
                        networkType = "unsigned",
                        powerVector = powers, verbose = 5)

sizeGrWindow(10,5)
par(mfrow = c(1,2))
par(mar=c(5,6,2,2))
cex1 = 1
plot(sft$fitIndices[,1], -sign(sft$fitIndices[,3])*sft$fitIndices[,2],
     xlab="Soft Threshold (power)",ylab="Scale Free Topology Model Fit,signed R^2",type="n",
```

```
        main = paste("Scale independence"),
        cex.lab = 1, cex.axis=1, cex.main = 1.5,cex=1.5 );
text(sft$fitIndices[,1], -sign(sft$fitIndices[,3])*sft$fitIndices[,2],
        labels=powers,cex=cex1,col="red");
abline(h=0.9,col="red")
plot(sft$fitIndices[,1], sft$fitIndices[,5],
        xlab="Soft Threshold (power)",ylab="Mean Connectivity", type="n",
        main = paste("Mean connectivity"),
        cex.lab = 1, cex.axis=1, cex.main = 1.5,cex=1.5)
text(sft$fitIndices[,1], sft$fitIndices[,5], labels=powers, cex=cex1,col="red")
graph2ppt(file='power-beta-value.pptx')

sft$powerEstimate

type = "unsigned"
nGenes = ncol(datExpr)
nSamples = nrow(datExpr)

net = blockwiseModules(datExpr, power = 7,
                       maxBlockSize = nGenes, minModuleSize = 30,
                       TOMType = "unsigned", corType = "pearson",
                       reassignThreshold = 0, mergeCutHeight = 0.25,
                       numericLabels = TRUE, pamRespectsDendro = FALSE,
                       saveTOMs =T,saveTOMFileBase = "FPKM-TOM",
                       loadTOMs = F,verbose = 3)
table(net$colors)

mergedColors = labels2colors(net$colors)
table(mergedColors)

expColor=t(numbers2colors(log2(datExpr+1),
                          colors=blueWhiteRed(100),naColor="grey"))
colnames(expColor)=rownames(datExpr)
sizeGrWindow(14,9)
plotDendroAndColors(net$dendrograms[[1]], mergedColors[net$blockGenes[[1]]],
                    "Module colors",
                    dendroLabels = F, hang = 0.03,
                    frame.plot = F,guideCount = 40,
                    addGuide = T, guideHang = 0.05,
                    cex.colorLabels = 1.5,
                    cex.lab = 1.5,cex.main = 2,cex.axis=1.3 ,
                    marAll = c(1, 8, 3, 0.1))
graph2pdf(file='genes-modules.pdf')
```

```r
modulelabels <-net$colors
moduleColors <-labels2colors(net$colors)
genetree <- net$dendrograms[[1]]
MEs <- net$MEs
save(modulelabels,moduleColors,genetree,MEs ,file = 'net_construction.Rdata')

table(pheno)
nGenes = ncol(datExpr)
nSamples = nrow(datExpr)
moduleColors <- labels2colors(modulelabels)
MEs0 = moduleEigengenes(datExpr, moduleColors)$eigengenes
MEs = orderMEs(MEs0)
moduleTraitCor = cor(MEs,pheno, use = "p")
colnames(MEs)
modules <-MEs
moduleTraitPvalue = corPvalueStudent(moduleTraitCor, nSamples)

file.remove('All_Gene_KME.txt')
for(module in substring(colnames(MEs),3)){
  #if(module == "grey") next
  ME=as.data.frame(MEs[,paste("ME",module,sep="")])
  colnames(ME)=module
  datModExpr=datExpr[,moduleColors==module]
  datKME = signedKME(datModExpr, ME)
  datKME=cbind(datKME,rep(module,length(datKME)))
  write.table(datKME,quote = F,row.names = T,append = T,file = "All_Gene_KME.txt",col.names = F)
}

for(module in substring(colnames(MEs),3)){
  if(module == "grey") next
  ME=MEs[,paste("ME",module,sep="")]
  dev.off()
  #p(paste("wgcna.", module, ".express.barplot.png", sep=""),height = 700,width = 900)
  par(mfrow=c(2,1),mar=c(0.3,5.5,3,2))
  da=t(scale(datExpr[,moduleColors==module]))
  colname <-colnames(da)
  plotMat(t(scale(datExpr[,moduleColors==module])),
          clabels=colname,cex.lab = 1.5,
          rlabels=F,cex.main=2,cex=2,cex.axis=1.5)

  par(mar=c(5,5,0,0.7))
  barplot(ME,col=module,main="",cex.main=2,ylab="Eigengene Expression",
          xlab=paste(module, "Module"),
          cex.lab = 1.5, cex.axis=1.5, cex.main = 2,cex=2)
```

```
    graph2png(file=paste(module, '.express.barplot.png', sep=""))
    graph2pdf(file=paste(module, '.express.barplot.pdf', sep=""))
}
dev.off()


modNames = substring(names(MEs), 3)
geneModuleMembership = as.data.frame(cor(datExpr, MEs, use = "p"));
MMPvalue = as.data.frame(corPvalueStudent(as.matrix(geneModuleMembership), nSamples))
names(geneModuleMembership) = paste("MM", modNames, sep="");
names(MMPvalue) = paste("p.MM.", modNames, sep="")

nGenes = ncol(datExpr)
intmodules <- modNames
nGenes = ncol(datExpr)
nSamples = nrow(datExpr)
geneTree = net$dendrograms[[1]]

load('WGCNA/FPKM-TOM-block.1.RData')
TOM=as.matrix(TOM)
genename = colnames(datExpr)
modNames
inModule = (moduleColors=="pink"| moduleColors=="greenyellow" | moduleColors=="black")
table(inModule)
table(moduleColors)
modgenename = genename[inModule]
modTOM = TOM[inModule, inModule]
dimnames(modTOM) = list(modgenename, modgenename)
cyt = exportNetworkToCytoscape(modTOM,
                               edgeFile = "CytoscapeInput-edges-0.02-selected-modules.txt",
                               nodeFile = "CytoscapeInput-nodes-0.02-selected-modules.txt",
                               weighted=TRUE,threshold=0.02,
nodeNames = modgenename, nodeAttr = moduleColors[inModule])
```

GENIE3

```
rm(list = ls())
options(stringsAsFactors = F)

library(dplyr)
library(export)
library(GENIE3)
```

```
fpkm <- read.table('pepper_fpkm.txt',row.names = 1,header = T,sep = '\t')
fpkmmad <- apply(fpkm,1,mad)
exprMat <- fpkm[apply(fpkm,1,mad)> 0.5,]
module <- read.table('WGCNA/All_Gene_KME.txt',row.names = 1,header = T,sep = '\t')

resdata <- merge(as.data.frame(exprMat), as.data.frame(module),    by='row.names',sort=F)
resdata <- resdata[,-20]
rownames(resdata) <- resdata[,1]
exprMatr <- resdata[,-1]
head(exprMatr)

data <- read.csv('WGCNA/pepper-TF_anno.csv')
rownames(data) <- data$Gene.ID
data <- data[,-c(1,3,4)]
res <- merge(as.data.frame(exprMatr), as.data.frame(data), by='row.names',sort=F,all=T)
res0 <- merge(as.data.frame(exprMatr), as.data.frame(data), by='row.names',sort=F,all=F)
write.csv(res,file = 'module-gene_TF.csv')

exprMatr0 <- read.csv('WGCNA/module-gene_TF.csv',row.names = 1)
dim(exprMatr0)

exprMatr0[661,]
exprMatr0[662,]
regulators0 <- c(1:661)
weightMat0 <- GENIE3(as.matrix(exprMatr0[,1:18]),
                      regulators =regulators0 ,
                      targets = NULL,
                      treeMethod = "RF",
                      K = "sqrt",
                      nTrees = 1000,
                      nCores = 10,
                      verbose = T)
dim(weightMat0)

linkList0 <- getLinkList(weightMat0)
dim(linkList0)
head(linkList0)
linkList01 <- getLinkList(weightMat0, reportMax=10000)
write.table(linkList01,file = 'GRN-all.12442-top10000links.txt',
            sep = "\t", row.names =F, col.names = TRUE)
```
